# Improving Diversity in Recruitment: Lessons Learned During the REACH Pregnancy Circles Pilot Trial

**DOI:** 10.1111/hex.70300

**Published:** 2025-05-21

**Authors:** Octavia Wiseman, Sidera Tahir, Christine McCourt, Anita Mehay, Helliner Robinson, Kade Mondeh, Lorna Sweeney, Meg Wiggins, Mary Sawtell, Angela Harden, the REACH Research Team

**Affiliations:** ^1^ City St George's University of London, Centre for Maternal and Child Health London UK; ^2^ University of East London, The Institute of Health and Human Development (IHHD) Water Lane London UK; ^3^ Barts Health NHS Trust, Royal London Hospital Whitechapel London UK; ^4^ Institute of Education University College London London UK

**Keywords:** group antenatal care, maternity, migration, patient involvement, recruitment, reduced inequalities, research participation

## Abstract

**Introduction:**

Our ability to address inequities in health outcomes is hampered by the under‐representation of underserved groups in research. Research exploring this topic has focused on observational studies in the American context. This is a pivotal concern for maternity research in the UK as perinatal outcome variables vary by ethnicity, socioeconomic and linguistic background. This paper reports the findings of an analysis of the diversity achieved by different recruitment strategies used within a feasibility study and pilot trial of group antenatal care (Pregnancy Circles).

**Method:**

A pilot randomised controlled trial involved implementation of Pregnancy Circles across three maternity services in an area of high ethnic, socioeconomic and linguistic diversity. Following findings of high ethnic diversity but low levels of educational and linguistic diversity amongst participants recruited in our prior feasibility study, equity‐informed strategies were put into place to attempt to increase recruitment diversity in the pilot trial, addressing organisational barriers (additional language support); attitudinal barriers (staff training to counteract recruitment bias) and practical barriers (extending the recruitment period to reach women accessing care late). Women who declined participation were invited to complete a short anonymous questionnaire covering demographic details and reasons for declining. The demographic characteristics of participants in the feasibility and pilot studies, and the pilot study decliners, were compared using descriptive statistics and free‐text reasons for declining were analysed thematically.

**Results:**

The targeted recruitment processes were successful in widening the diversity of participants in this study, in particular for women with limited English proficiency and low educational achievement. Nevertheless, comparison of participants to those who declined showed some barriers persisted. The most common reason to decline was lack of time, most commonly due to caring responsibilities, and this was more likely to be cited by ethnically minoritized women.

**Conclusion:**

Recruitment plans focused on widening diversity can be effective but are likely to require additional resources such as funding longer recruitment periods or interpreting services. The gendered nature of maternity research poses particular challenges, and our study suggests that addressing barriers such as those around childcare would enhance the recruitment of socio‐economically deprived and minoritized women.

**Patient or Public Contribution:**

Our study team included two service user representatives as co‐investigators, feeding into all aspects of the study. The focus of the work reported here was to increase the participation of underserved communities in the pilot trial and to inform the Pregnancy Circles RCT, to enable them to contribute their data and lived experience to the findings and evaluation of this intervention.

**Trial Registration:**

Due to an administrative oversight, trial registration for this pilot trial was applied for during the 6‐week recruitment period, rather than before recruitment commencing (ISRCTN66925258. Retrospectively registered 3 April 2017). Registration occurred before programme intervention, outcomes and process data collection and all data analysis

**Abbreviations**
gANC: group antenatal care
LEP: limited English proficiency
PC: pregnancy circles
REACH: research for equitable antenatal care and health

## 1 Introduction

This article reports the findings of an analysis of the diversity of recruitment to health studies conducted within the context of a maternity care pilot trial, Pregnancy Circles [1]. The objective was to learn lessons about how to optimise recruitment of women from a diverse range of backgrounds into maternal health research, within the context of a trial of a midwifery care model intended to enhance access and experiences of antenatal care for underserved groups. In this context, ‘underserved’ was defined as socio‐economically disadvantaged, racially minoritised and linguistically excluded.

### 1.1 Involving Underserved Groups in Research

Our ability to address disparities in health outcomes is hampered by the underrepresentation of women and underserved groups in clinical research [2]. A recent observational study of NIHR‐funded research found that areas with the highest burden of disease have the lowest number of patients taking part in research [3]. This is a pivotal concern for health research as many outcome variables vary by gender, ethnicity and socioeconomic background [4]. Testing interventions on participants who do not reflect the target population not only has ethical implications, but can result in less effective treatments and reduce trust [3, 4, 5]. Ethnic disparities in health outcomes were brought into sharp relief during the COVID‐19 pandemic and are a focus of concern in maternity care [6]. It is important for studies to prioritise broadening participation to develop appropriate, effective and trusted interventions, and this is especially relevant for Pregnancy Circles, a model of care whose mechanisms of effect are predicated on interpersonal relationships which can be affected by a range of social factors [7].

A range of barriers to recruiting socio‐economically disadvantaged and ethnically minoritised women in research have been identified, including in maternity research [2, 8]. Women from underserved communities may lack familiarity with research or have concerns about potential harms or even exploitation [9]. The financial burden of participating in research (e.g. paying for travel or childcare) may have a disproportionate impact on economically deprived populations [2]. While financial compensation may increase participation it also raises ethical issues as poor participants may not feel they can afford to decline [10]. Competing responsibilities such as work and childcare are an important issue for ethnically minoritised women, whose families may play an integral role in their decision‐making [11]. A crucial factor for minoritised groups in deciding whether to join a research study is whether participants trust the research/clinical team [9] but in recent years, the socio‐political context in the UK has limited some migrants' rights and access to healthcare, increasing fear and mistrust of health services [12]. In the UK, a lack of funding for interpreting and translation services has been identified as a major reason for the underrepresentation of diverse ethnic groups in research [13]. Misinterpretation and misunderstanding of English‐language printed materials are common and, even with access to interpreters, participants may feel their language and literacy skills will prevent them from actively contributing to the study, or they may have concerns about privacy when using interpreters [14].

Suggested approaches to recruiting more diverse cohorts in research include hiring researchers from the same ethnicity, using simple, nontechnical language and engaging with local communities [2, 8, 14, 15].

In 1993, the American government passed legislation to prioritise the inclusion of diverse groups in clinical research [16]. This triggered many studies exploring barriers to participation, but only a slight improvement in actual participation by African‐Americans and Asian‐Americans, despite evidence that they are keen to engage in clinical research [2]. In 2016, the UK National Institute for Health Research (NIHR) published recommendations to address gender inequality in medical research in the UK, but it was not until 2020 that they launched INCLUDE guidance to support the involvement of under‐served groups in research [17]. More recently, Trialforge has built on this study to produce frameworks and practical approaches to support inclusion in trials [15]. A recent systematic review exploring factors influencing ethnically minoritized women's participation in maternity research [8] found that 10 of the 13 studies identified took place in America and almost all explored recruitment to observational studies. They conclude that more research is needed exploring this group of women's participation in trials, especially outside the US context.

### 1.2 The Pregnancy Circles Study

The NIHR‐funded Research for Equitable Antenatal Care and Health (REACH) Pregnancy Programme aimed to explore equity in access to, and experience of, antenatal care and part of the programme focused on whether group antenatal care (gANC) could improve experiences and outcomes for women living in areas of high socioeconomic, ethnic and linguistic diversity in the UK. RCTs are important as they have the potential to change healthcare delivery locally and internationally. It is widely acknowledged that women from socio‐economically disadvantaged and ethnically minoritised groups report more negative experiences of maternity care despite having potentially greater social and medical needs [18]. They are at significantly higher risk of poor maternal and neonatal outcomes, with intersectionality between gender, economic disadvantage and ‘a constellation of biases’, exacerbating risk [6, 19]. There is an urgent need to understand how to engage women and birthing people most in need in maternity health research.

Group antenatal care combines clinical care, information‐sharing and social support. Originally developed in America under the name CenteringPregnancy® [20], various models of gANC have been implemented in Europe, Africa, Iran, India and Australia. A Cochrane review in 2015 found that gANC increases women's satisfaction with care without adverse outcomes and a systematic review of trials and cohort studies focused on high‐risk and vulnerable groups (including African Americans, teenage mothers, low‐income women) reported improvements in preterm birth, breastfeeding, smoking cessation, psycho‐social outcomes and attendance at antenatal care [21, 22]. The REACH team developed a Centering‐based model of gANC (Pregnancy Circles) for implementation within the UK National Health Service (NHS) where two midwives provide care for 6‐12 women due within a month of each other. The 2‐h sessions focus on group activities and women‐led discussions, replacing traditional one‐to‐one appointments. Women are taught to carry out their own health checks (blood pressure and urine testing) and receive a brief private check with the midwife within the group space [23]. There is relational continuity of the facilitators and the group participants through the pregnancy and for one postnatal follow‐up group session.

### 1.3 Feasibility Study

Early exploratory and Patient and Public Involvement (PPI) work identified that women preferred the idea of culturally and linguistically mixed groups. The Pregnancy Circles feasibility study (2015–16) was designed to test the acceptability of Pregnancy Circles, including in mixed groups. Four ‘test’ Pregnancy Circles were run across three maternity services in areas of inner London with a high level of diversity. Women whose babies were due within the 4‐week estimated delivery date range and geographical area of their local Pregnancy Circles were suitable to be recruited, regardless of gestation, obstetric risk profile, socioeconomic, linguistic or cultural background. Women were recruited during their booking appointment (8‐12 weeks) and the only exclusions, for ethical reasons, were women with an impaired ability to provide consent (women under 16 or with a documented learning disability) and women who fit the criteria for referral to local specialist teams (i.e. the ‘vulnerable team’ caring for women with substance abuse or social services involvement or the ‘young parents’ team) to ensure that nobody would miss out on specialist care by taking part in our study. These services, at the time of the study, employed bilingual health advocates (BHAs) able to provide a blend of language and cultural interpretation for maternity clients, including during their booking appointment if needed. Twenty‐four participants were recruited to the feasibility study of which 63% (*n* = 15) were born outside the UK. Of these, 38% (*n* = 9) were Asian or British Asian (predominantly of Bangladeshi origin) and only 21% (*n* = 5) were White British. The study found that being part of a Pregnancy Circle of mixed parity, ethnicity, religion and culture was particularly valued by women, challenging previously accepted normative beliefs [24]. Early concerns from managers and commissioners about whether ethnically minoritised women (in particular Muslim women) would want to receive care in a group were not borne out in practice [25].

While the participants in the feasibility study were more ethnically diverse than often found in health research, on closer examination some limitations became clear. Despite the ethnic diversity, there was little educational or linguistic diversity: all of those recruited were either native speakers or spoke English ‘well’. In addition, all but one participant had received higher education beyond 18 years of age, more than half to degree level or beyond. Nobody who had left full‐time education at 16 years or below had been recruited.

The team theorised that the lack of diversity on these dimensions may have been due to a combination of practical, attitudinal and organisational barriers. Practical barriers included lack of childcare which was anticipated to affect some groups more than others [11] and women with socially complex lives being more likely to book late for their pregnancies, thus missing the recruitment window [26]. The main organisational barrier was perceived to be insufficient access to language support, as BHAs were not always available in the booking clinic when recruitment took place. Attitudinal barriers included women from some backgrounds being less familiar with, or wary of, research, as well as bias on the part of service providers about who was, or was not, ‘suitable’ for group care so that at times research midwives or nurses did not approach women who they assumed would not be suitable [25]. It is common for women with social risk factors to report paternalistic attitudes and discrimination in maternity services [18].

This paper reports on the techniques put in place by the REACH team to recruit a more diverse and representative group of women to the pilot trial of Pregnancy Circles, and the impact observed on the diversity of participants. Other findings from the pilot trial have been reported elsewhere [1].

## 2 Methods

This article presents our analysis of the data gathered in the relation to diversity of recruitment to our research on Pregnancy Circles. Our analysis of the experiences of women with limited English proficiency who participated in the study is reported elsewhere [27]. We asked

- How do targeted recruitment techniques affect the diversity of the sample achieved in the Pregnancy Circles pilot trial when compared to the feasibility study?
- What reasons were given for declining to participate in the pilot study, and were these related to ethnicity, educational level or language skills?

### 2.1 Setting

The pilot trial took place in the same UK acute NHS Trust as the feasibility study. Three Pregnancy Circles were implemented between December 2016 and March 2018.

### 2.2 Recruitment

To maximise the possibility of recruiting more diverse participants to the pilot trial than was achieved in the feasibility study, the research team decided to use an equity lens when designing recruitment techniques for the pilot study. Three areas emerged as important to address, informed by a literature review and our findings in the feasibility study: organisational, attitudinal and practical barriers, as discussed next.

#### 2.2.1 Recruitment Strategy 1: Addressing Organisational Barriers

Organisational barriers were considered to be those where the structure of care delivery might act as a barrier to being offered participation in the study. The main organisational barrier we identified was insufficient or uneven language support. The research team presented the Pregnancy Circles study to the Bilingual Health Advocates (BHA) team to familiarise them with Pregnancy Circles in advance of recruitment starting so that they felt confident explaining the trial to potential participants and understood that women who needed language support were suitable for inclusion. BHAs are drawn from the local community and employed by the maternity services to act as interpreters and provide cultural mediation. The study also funded a telephone interpreting service called Language Shop which could be used if BHAs or bilingual researchers were not available. On the advice of the local research team, we did not focus resources on translating written materials due to the wide range of languages spoken locally: one site had 104 languages and their experience was that verbal explanation was more important than written materials in recruitment.

#### 2.2.2 Recruitment Strategy 2: Addressing Attitudinal Barriers on the Part of Both Staff and Potential Participants

Evidence from the feasibility study and the wider literature [25, 28] has shown that staff can act as gatekeepers, limiting access to services for underserved groups. In the case of Pregnancy Circles, we were aware of midwives making assumptions about which women were ‘suitable’ or not for group care. The research team sought to address staff bias by hosting a lunch at each site for the booking midwives to familiarise them with the study, clarify the inclusion criteria and stress that ethnicity, culture, religion, socioeconomic background and English‐language proficiency should not be a barrier to participation. We also sought to address lack of trust on the part of potential participants [9] by using bilingual researchers who spoke their language wherever possible during recruitment and follow‐up phone calls.

#### 2.2.3 Recruitment Strategy 3: Addressing Practical Barriers to Participation

Recruitment strategies must be tailored to ensure equity [5]. We examined our recruitment processes and identified that limiting recruitment to 8–12 weeks gestation, as we did in the feasibility study (since most women book their pregnancies by 10 weeks gestation) would exclude women who book late for care, including some ethnically minoritised women, young mothers and women with more than four children [29]. To avoid this practical barrier to participation in the pilot trial the recruitment period was extended to 20 weeks gestation, including face‐to‐face recruitment of women at their 12‐week and 20‐week scans as well as at bookings. Evaluation of the feasibility study had suggested that women could join a Pregnancy Circle at the second session (25 weeks gestation) without disrupting the group dynamic [24]. In addition, the REACH team agreed to fund a creche at each site to support women with toddlers to participate in the pilot trial.

### 2.3 Inclusion and Exclusion Criteria

The sample size for the pilot trial was 72, with up to 24 women planned to be recruited in each maternity service, half being randomised to the intervention (Pregnancy Circles) and half to the control (traditional antenatal care consisting of 20‐min individual appointments) [30]. All women booking with the service whose geographical location and expected delivery date fitted with the planned Circle were eligible. As with the feasibility study, the only exclusions were women with an impaired ability to consent or who met the requirement for referral to a specialist team. Participants were offered a £10 voucher to thank them for their time for each of the three questionnaires they filled in for the pilot trial.

### 2.4 Measuring Diversity

For the feasibility and pilot trial, we measured a range of factors linked to greater risk of adverse pregnancy‐related outcomes to measure diversity in participants, including ethnicity [31, 32, 33], limited English proficiency [34], age [35], migration status [36] and educational level [37, 38]. Parity was measured to understand whether having responsibility for children affected women's ability to attend longer antenatal appointments. Measuring risk based on innate or situational factors is complex and potentially stigmatising and it is important to acknowledge that adverse outcomes in these groups are not intrinsic but generally related to the social and institutional context within which care is delivered [39]. For the 5‐min decliner interview, we wanted to minimise the burden of data collection for women who had, after all, not consented to participate. We therefore limited the demographic information collected to factors we felt were most indicative of social diversity: ethnicity, English language proficiency, educational level and parity. We report on these in this paper. Table 1 outlines the demographic data collected in the feasibility study (participants) and pilot trial (participants and decliners):

**Table 1:** Demographic data collected in the pregnancy circles feasibility and pilot trial studies.

Demographic details	Definition	Feasibility study participants	Pilot trial participants	Pilot study decliners
Ethnicity	1. **White** (British, Irish or Other) 2. **Black** (African, Caribbean, Black British or Mixed) 3. **Asian** (Indian, Pakistani, Bangladeshi, Chinese, other Asian, Asian British or Mixed) 4. **Other** (Arab, other ethnic group or other mixed background)	Y	Y	Y
Born in the UK	1. Yes 2. No	Y	Y	N
Age	What is your age? 1. 16–19 2. 20–25 3. 26–35 4. 36 years or more	Y	Y	N
English proficiency^a^	1. English is my main language 2. Very well 3. Well 4. Not well 5. Not at all	Y	Y	Y
Educational qualification	1. No qualifications 2. GCSE or similar (16 years) 3. A‐Level or similar (18 years) 4. Degree or postgraduate	Y	Y	Y
Parity	How many babies have you given birth to? 1. None, this will be my first 2. 1 baby 3. 2–3 babies 4. 4 or more	Y	Y	Y

^a^ 4 and 5 were considered to have limited English proficiency (LEP).

### 2.5 Data Collection

Women who consented to take part in the study had their demographic information collected as part of a baseline data collection sheet (feasibility study) or the baseline questionnaire (pilot study). Those who declined were offered the opportunity to answer a brief (5‐min) anonymous questionnaire which included four demographic questions (outlined above) and free text asking for their main reasons for declining to participate. Consent was implied if they agreed to fill in the questionnaire. Women could fill in the questionnaires on paper by themselves or with the support of a family member, researcher or interpreter, according to their preference.

### 2.6 Data Analysis

Data about decliners were not collected in the feasibility study. Demographic data from both feasibility and pilot trial studies were analysed in SPSS V22 using descriptive statistics to compare participants in both studies, and to compare participants to decliners in the pilot trial. Free‐text reasons for declining the pilot study were uploaded to NVivo11 and thematic analysis was used to identify different narratives for not participating [40].

### 2.7 Ethical Approval, Consent and Data Protection

Research Ethics Committee (REC) ethical approval for this study was granted (REC 16/NS/0090), and data protection processes were followed as outlined in the pilot trial protocol [23].

## 3 Results

Two‐hundred and forty women were approached to participate, of whom 74 were recruited to take part in the pilot trial. Of the 165 who declined, just under half (*n* = 73) agreed to fill in the questionnaire about their reasons (‘decliners’). No information was collected for the other 95 women. The research team reported that recruiting women with LEP required significantly more time and effort, particularly telephone follow‐up using interpreters for those who chose to take more time to consider whether to take part in the trial.

Despite efforts to provide a funded creche for the Pregnancy Circles this turned out not to be possible in any of the sites due to venue restrictions, so free childcare could not be offered at the point of recruitment.

The demographic characteristics of participants and decliners for the pilot study are outlined in Table 2, alongside those of participants in the feasibility study. Demographic data was more complete for feasibility and pilot trial participants who had consented to take part in the studies, compared to the decliner's questionnaire (ethnicity 70%; education 86%; parity 80%; English proficiency 91%). Missing data were excluded when calculating percentages.

**Table 2:** Demographic characteristics of the pregnancy circles feasibility study participants, pilot trial participants and pilot trial decliners.

Characteristics		Feasibility study, *N* (%) (total = 24)	Pilot trial participants, *N* (%) (total = 74)	Pilot trial decliners *N* (%) (total responses = 64)
Education	No qualifications	0 (0)	3 (4.1)	5 (7.8)
	GCSE/vocational (16 years)	0 (0)	22 (29.7)	16 (25.0)
	A‐level or similar (18 years)	2 (8.3)	10 (13.5)	11 (17.2)
	Degree/post‐grad	22 (91.6)	39 (52.7)	32 (50.0)
	Missing	0	0	9
Ethnicity	White British/White Other	9 (37.5)	28 (37.8)	23 (42.6)
	Black, Black mixed or Black British	10 (41.6)	7 (9.5)	3 (5.6)
	Asian, Asian mixed or Asian British	3 (12.5)	33 (44.6)	28 (51.9)
	Mixed/Other	2 (8.3)	5 (6.8)	0 (0)
	Missing	0	0	19
English language proficiency	English spoken well/very well	24 (100)	62 (84.9)	48 (71.6)
	Speaks English not well	0 (0)	10 (13.7)	15 (22.4)
	I do not speak any English	0 (0)	1 (1.4)	4 (6.0)
	Missing	0 (0)	1	6
Parity	Primipara	11 (47.8)	39 (52.7)	20 (33.3)
	Multipara	12 (52.2)	35 (47.3)	40 (66.7)
	Missing	1	0	13

### 3.1 Parity

In both the feasibility and the pilot trial, roughly equal numbers of women having their first baby (primipara) and women having a second or subsequent baby (multipara) were recruited. However, the decliner interviews for the pilot study highlighted that this apparent equity was misleading: more multiparous women were approached to take part and 67% declined compared to 33% of primiparas. In addition, multiparas were more likely to decline the more children they had.

### 3.2 Education

Twenty‐five women who had received no education beyond 16 years were successfully recruited to the pilot trial compared to none in the feasibility study, increasing the educational diversity of participants.

### 3.3 Language Proficiency

Eleven (15%) of the participants recruited to the pilot study had limited English proficiency (LEP: i.e. they spoke ‘no’ English, or ‘not well’), compared to none in the feasibility study. Although only one of the decliners cited language as a barrier to participation in the pilot study, roughly a third of women with LEP approached agreed to participate, compared to over half of the women who spoke English well/very well, suggesting that barriers to participation still remained for women with LEP, although the strategies did address these to some degree.

### 3.4 Ethnicity

In the pilot study there was no difference in the proportion of ethnically minoritised women who chose toto participate or decline, and no women cited race, ethnicity or religion as a reason for declining. The largest group recruited to the pilot trial were Asian (45%) and the proportion of White British/White Other who consented to participate remained the same across both studies (38%).

### 3.5 Reasons for Not Participating in the Pilot Trial

**Figure 1:**
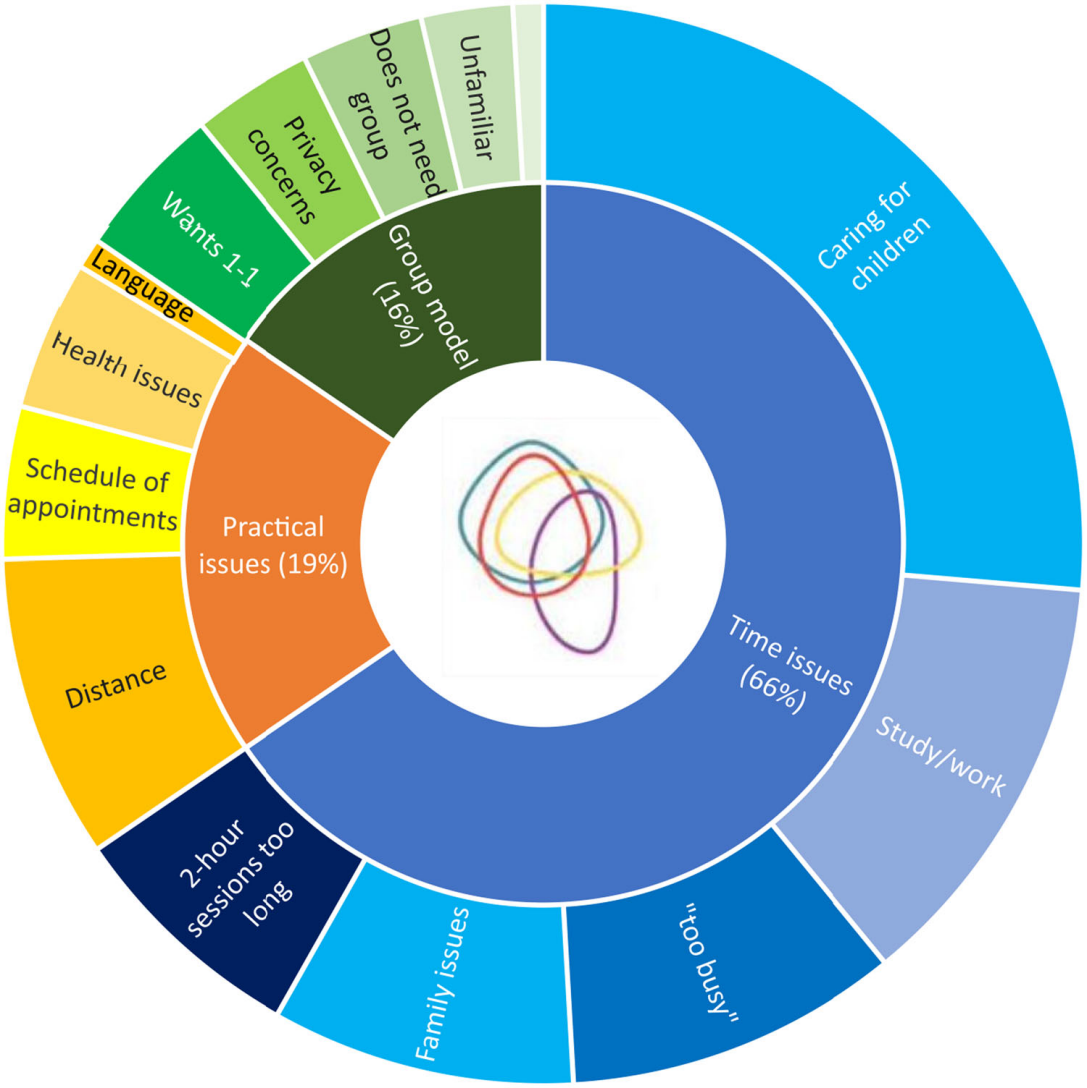
Reasons for declining Pregnancy Circles pilot study.

Most of those who responded to the ‘decliner’ form gave one primary reason for declining, with just over a third giving two reasons (e.g., caring responsibilities and not being able to get time off work, or not having childcare and having lots of other appointments). Reasons for deciding not to take part in the pilot trial were coded into categories and organised under three main themes: time issues, practical issues and issues related to the model of care (Figure 1). Nobody mentioned the randomisation element as a reason for declining.

### 3.6 Practical Issues: Time

Not having enough time or being ‘too busy’ to attend the 2‐h sessions was the most common reason given for declining (65.5%): a fifth of those who gave this reason specified that they could not get time off work (more common with primiparas and women from a White Other background):

Work commitments – I'm self‐employed so not straightforward taking time off.

[White, English speaker]

Just over half cited informal caring responsibilities, primarily childcare. Nearly a third said they would have participated if there had been a creche or if children were welcome in the session. Seventy‐three percent of South Asian decliners cited caring responsibilities compared to 28% of White British or White Other decliners. Most women with LEP who declined reported problems with childcare and getting time off work.

Woman's job, she works all days of the week in a restaurant, including Saturdays. Can't be absent 2 h.

[White Other, LEP – written by research midwife]

Great idea, personally likes it and if she didn't have other commitments [childcare] she'd like to have taken part. She likes the idea of talking to other women.

[Pakistani, English speaker – written by research midwife]

Longer midwifery appointments are a key characteristic of the Pregnancy Circles model of care, but only 10% of those who cited time issues said that they felt that 2 h was ‘too long’. Most cited not having ‘enough time’ to attend, and this affected ethnically minoritized women disproportionately.

### 3.7 Practical Issues

A quarter of women who declined did so for other practical reasons, primarily the geographical distance or timing of the Circle.

Far away from [home], she is recently arrived and does not know the buses.

[Pakistani, LEP]

Five women declined due to health issues (anxiety over complex pregnancies or having too many appointments) and three of whom had LEP.

Because of [medical] condition does not feel appropriateto be in a group

[White Other, LEP]

Miscarriage in the past – needs bed rest so 2 h would be difficult

[Pakistani, LEP]

She has lots of appointments during pregnancy and feels this may be too much

[Pakistani, English‐speaker]

### 3.8 Issues Related to the Model of Care

A few women declined because they were not familiar with the model of care (*n* = 3):

Haven't heard of it before – If heard before, received in post, maybe more likely to accept?

[White British, English speaker]

Five felt they did not need or could contribute to a group for a variety of reasons:

I already have a strong network of support

[White British, English speaker]

Doesn't feel she needs circles as this is not her first baby

[Bangladeshi, LEP]

I feel I will be able to be of more help in a circle if it was my 2nd child

[Black African, English speaker]

Three women declined because they wanted their partner to be present at all appointments (the initial Circles session was planned to be women‐only, with the group deciding how often to invite partners thereafter). Only nine women (12.3%) declined because they did not like the idea of care in a group, five because they preferred one‐to‐one appointments, and four because they had concerns about privacy. This was more common among White British women compared to other ethnic groups, as one told us:

I don't really want to talk about my pregnancy.

[White British, English speaker]

## 4 Discussion

### 4.1 Impact of Targeted Recruitment Techniques

The enhanced recruitment techniques used in this pilot study included improving communication, addressing bias, enhancing language support and widening the recruitment window to capture late bookers. Although it is not possible to identify which elements had the most impact on recruitment, using an equity lens in designing recruitment techniques successfully increased the diversity of participants in the Pregnancy Circles pilot study compared to the feasibility study. In particular, women with LEP and those with lower educational achievements were recruited to the pilot study (making up 15% and 34% of participants, respectively) compared to none from either group in the feasibility study). Despite this, some barriers to participation remained, as data from the decliner interviews showed that multiparas and women with LEP were less likely to consent to the pilot study compared to women having their first baby or who spoke English well.

### 4.2 Reasons for Declining

The underrepresentation of ethnically minoritized groups in research cannot be assumed to be explained by a lack of interest in participation [2]. This study did not find that ethnic background per se was a barrier to participation in the Pregnancy Circles trial, confirming the findings of the feasibility study [24]. Several ethnically minoritized decliners mentioned that they would have liked to participate had they not faced practical barriers. By exploring women's reasons for not participating, we were able to understand (and potentially address) the barriers most likely to affect minoritized groups in this study.

The Pregnancy Circles intervention required participants to commit to longer antenatal appointments (2 h rather than 20 min), so ‘time issues’ was, not surprisingly, the most common reason given for declining, with caring responsibilities, primarily childcare, being cited most often. We identified intersectionality between gendered caring responsibilities and migrant status, with South Asian women and women with LEP more likely to have larger families and hence to give lack of childcare as their primary reason for not participating. While participants in the pilot trial were offered £10 compensation for their time for each of the three questionnaires, this would not have been sufficient to cover childcare costs.

Another common reason for declining due to ‘not enough time’ was work responsibilities. In the UK, pregnant employees are entitled to time off with full pay for antenatal appointments and parent education, including travel time but this is not always accessible [41]. Research by the Equalities and Human Rights Commission found that 77% of women report negative or discriminatory treatment at work during pregnancy [42]. In addition, paid time off is less available for women on low pay who are more likely to have part‐time or casual contracts.

This study identified gendered caring responsibilities and disempowerment (not being able to access their legal right to time for antenatal care) as significant barriers to participation, with these disadvantages disproportionally affecting women living with social complexity. It is widely acknowledged that being female is an independent barrier to participation in research [43], despite the fact that participation in trials has been shown to improve women's health [44]. The gendered nature of maternity poses particular challenges to researchers, and our study suggests that addressing gendered barriers, in particular around childcare and women's rights, would enhance the recruitment of socio‐economically deprived and minoritized women.

In the UK, more than one in four live births are to mothers who were born abroad and in our inner‐city setting, this proportion was much higher [45]. It is thus not surprising that enhancing language support was an effective tool to increase the recruitment of women with LEP. The literature suggests that more efforts in this direction (written materials in different languages; outreach to communities) might improve recruitment rates further [8, 11, 14, 15]. Including women with LEP in this study produced invaluable and novel insights into their experiences, feeding into future implementation and research [27].

Findings from this study informed the protocol for the full Pregnancy Circles randomised controlled trial [46], and the development of bespoke training for recruiters and facilitating midwives to address persistent barriers to recruitment and participation. As well as the techniques implemented in the pilot trial, changes to the conduct of the RCT included

- Childcare: we learned that implementing a creche is very challenging within NHS services, so the possibility of adapting the Pregnancy Circles model to enable women to bring their toddlers was integrated into facilitation training (the choice of whether they could manage this fell to individual services and facilitating midwives).
- Translation: The trial PIS included a statement in 6 languages saying that interpreting support was available (English, Urdu, Somali, Bengali, Romanian, Polish)
- Recruitment training: since recruitment for the full trial was carried out by site research teams rather than by the study research team, bespoke training was developed including discussions about bias, inclusion, accessing interpreters and information‐sharing about women's rights to access care.
- Sites were encouraged to include their Maternity and Neonatal Voices Partnership in their steering group, to enable service users to feed into local design and implementation.
- Emerging research into the impact of economic deprivation on perinatal outcomes meant that for the full trial, we collected additional data on participants' Index of Multiple Deprivation [33, 47].

### 4.3 Strengths and Limitations

Limitations of this study include the fact that we were unable to collect demographic data or reasons for declining from 95 (just over half) of those who decided not to participate in the pilot study. Because of resource implications, the diversity of the area and organisational issues, we were unable to explore the impact of other approaches to improving recruitment, in particular providing childcare, community engagement and producing written materials in diverse languages, which has been shown to support greater equity in participation [14, 15]. Data about women's perceptions was collected via a 5‐min pro‐forma, which limited the detail provided. This was a pragmatic decision taken to maximise key information while minimising the burden on pregnant women who had declined to take part in the pilot trial. Future research would benefit from including in‐depth interviews, which could shed light on the impact of different recruitment techniques on different groups' decisions about participation and adopting co‐creation recruitment strategies with the most underrepresented groups, although these may not be acceptable to some who have declined participation. Equally, relying solely on descriptive analysis rather than analytical methods may lead to incorrect interpretation of the findings. A larger study with robust sample size calculation and statistical analysis could test the findings of this exploratory study.

A strength of this study was our ability to observe the impact of equity‐informed approaches to recruitment in the same Trust and geographical area for the same intervention and to explore reasons for nonparticipation in a very diverse population. This study demonstrated both the effectiveness of these approaches in increasing participation by underserved groups, in particular women with LEP and low educational levels and, conversely, the fact that these are not sufficient to even the playing field for minoritized groups who face systemic barriers and complex lives, especially those with gendered responsibilities such as childcare. We also identified that the randomisation element of the pilot trial was not identified as a barrier to participation, perhaps because it was a social rather than a clinical intervention. The inclusive pilot trial design contributed internal validity and our findings suggest that focusing on equity when designing recruitment processes can broaden diversity. However, caution is needed before generalising these particular recruitment strategies to other contexts. In particular, our sample came from an area of high socioeconomic diversity, and more targeted processes may be needed to identify and recruit minority populations or groups with specific challenges such as impaired abilities or young parents.

## 5 Conclusion

Barriers to participation in research identified in our study overlap with barriers immigrant women face when accessing antenatal care [48]. We demonstrated that developing targeted recruitment processes which take into account organisational barriers (in this case focusing on access for women with limited English proficiency), professional bias, and practical challenges such as late booking, were successful in widening the diversity of participants in this study, although some barriers persisted.

Gendered issues, in particular caring responsibilities and a lack of paid time off for maternity care, were the most common reasons given for declining, and were more likely to be cited by ethnically minoritized women and those with LEP. INCLUDE guidelines recommend careful consideration of who a trial applies to when designing both the intervention and the study [15]. Our findings suggest that addressing gendered barriers is likely to increase socioeconomic, educational, ethnic and linguistic diversity in maternity research. Further research is needed to explore intersectional challenges to participation in research for particular groups and the impact that additional techniques such as community engagement, translated materials and childcare provision would have on recruitment.

Future research studies, and study funders, must be mindful of the additional resources needed to successfully recruit more diverse participants to this study, including funding a longer recruitment period and the cost of translation and interpreting services.

## Author Contributions

**Octavia Wiseman:** conceptualisation, investigation, writing – original draft, methodology, visualisation, data curation, formal analysis. **Sidera Tahir:** investigation, writing – review and editing, methodology, formal analysis, data curation. **Christine McCourt:** conceptualisation, writing – review and editing, supervision, formal analysis. **Anita Mehay:** writing – review and editing, formal analysis. **Helliner Robinson:** investigation, writing – review and editing, project administration. **Kade Mondeh:** methodology, writing – review and editing. **Lorna Sweeney:** project administration, writing – review and editing, conceptualisation, methodology. **Meg Wiggins:** conceptualisation, methodology, writing – review and editing. **Mary Sawtell:** conceptualisation, methodology, writing – review and editing. **Angela Harden:** conceptualisation, methodology, writing – review and editing, funding acquisition.

## Ethics Statement

Research Ethics Committee (REC) approval was granted for this study (REC 16/NS/0090).

## Consent

Participants in the pilot study gave written, informed consent for their demographic data to be collected. Those who declined to participate were offered the opportunity to answer a brief (5‐min) anonymous questionnaire. Consent was implied if they agreed to fill in the questionnaire. No identifiable data is included in this article and no material from other sources has been reproduced herewith.

## Conflicts of Interest

The authors declare no conflicts of interest.

## Data Availability

The authors have nothing to report.
